# Emission of Photons by Near-Infrared PbS Quantum Dot Nanocrystals for a Large Diameter Range

**DOI:** 10.1021/acs.jpcc.6c00396

**Published:** 2026-05-26

**Authors:** Andreas S. Schulz, Christian Blum, G. Julius Vancso, Jurriaan Huskens, Willem L. Vos

**Affiliations:** † Complex Photonic Systems (COPS), Faculty of Science and Technology, 3230 University of Twente, P.O. Box 217, Enschede 7500 AE, The Netherlands; ‡ Molecular Nanofabrication (MNF), MESA+ Institute, 3230 University of Twente, P.O. Box 217, Enschede 7500 AE, The Netherlands; § Materials Science and Technology of Polymers (MTP) and Sustainable Polymer Chemistry (SPC), MESA+ Institute, 107537 University of Twente, P.O. Box 217, Enschede 7500 AE, The Netherlands; ∥ Nanobiophysics (NBP), Faculty of Science and Technology, 207105 University of Twente, P.O. Box 217, Enschede 7500 AE, The Netherlands

## Abstract

In pursuit of the functionalization of 3D silicon nanophotonics with optically active building blocks, we investigate, as candidates, near-infrared (NIR) emitting semiconductor quantum dot (QDot) nanocrystals. We present continuous-wave and time-resolved spectral emission of different commercially obtained lead sulfide (PbS) quantum dots with considerable 2-fold variations in diameters, with two different cappings—polyethylene glycol (PEG) and oleic acid (OA)—that are suspended in three different solvents: water, chloroform, or toluene. The emission spectra reveal maxima at different photon energies, as set by the nanocrystals’ average diameters. Time-resolved emission histograms reveal varying amounts of nonsingle exponential decay, all of which are successfully modeled with a log-normal distribution of decay rates. We observe, for quantum dots with OA surface groups, that the most frequent decay rate varies slightly with photon energy and concomitant quantum dot diameter. The rates agree with a two-level exciton model, especially if we assume the transition dipole moment to vary proportionally to the quantum dot diameter, but not with an atomic-like dipole emitter. For quantum dots with PEG surface groups, the most frequent decay rate decreases remarkably beyond photon energies 8000 cm^–1^ or conversely nanocrystal diameters below 4.4 nm. A hypothesis for this observation is that the protonation of the terminal amine groups of the thiol–PEG–NH_2_ ligands generates an enhanced local electric field, which enhances the quantum-confined Stark effect (QCSE). This leads to a greater spatial separation of the electron and hole wave functions and thus reduces the radiative rate, particularly in smaller dots. Other mechanisms, such as quenching, solvent polarity, solvent resonance (vibrational or electronic), nanoparticle acoustics, and electron or hole wave function trapping, are also evaluated. As a preliminary concrete step toward silicon photonics, we study quantum dots dip-coated on a silicon surface and observe that the emission rates are markedly increased 5- to 10-fold, which is attributed to the higher dielectric function near the silicon surface compared to suspensions.

## Graphical abstract

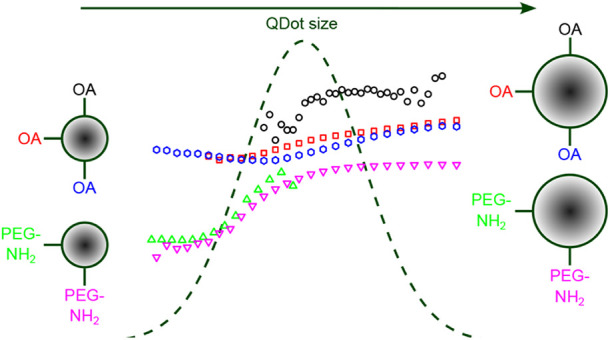

## Introduction

There is a lively scientific and technological interest in the use and application of semiconductor quantum dots as light emitters, even down to single photons.^1–3^ Many efforts are geared at the quantum dots in the near-infrared spectral range^4^ since such quantum dots are compatible with many applications that build on silicon nanophotonics, and on photonic telecommunications from long-range down to short-range on-chip data processing.^5–9^ Quantum dots are semiconductor crystals of nanometer dimensions with characteristic optical properties that are determined by their size.^1,2,10^ Since their diameters are comparable or even smaller than the extent of the electron and hole wave functions, the wave functions are quantum confined, revealing characteristic discrete energy levels, as opposed to the continuum levels in bulk semiconductors,^1,11–15^ as illustrated in Figure 1. The properties of electrons (at high energies in the conduction bands) and holes (at low energies in the valence bands) are effectively described by the well-known quantum mechanical particle-in-a-box problem. Due to quantum size confinement, the electron levels are shifted up with decreasing quantum dot diameter and the hole levels shifted down, leading to an increase (or blue shift) of the exciton energy, see Figure 1. These properties have allowed to synthesize light emitters with a desired range of photon energies, which has contributed to the great popularity and proliferation of quantum dot nanocrystals,^1,11,12,14^ culminating in the 2023 Nobel prize to Bawendi, Brus and Ekimov.

**Figure 1:**
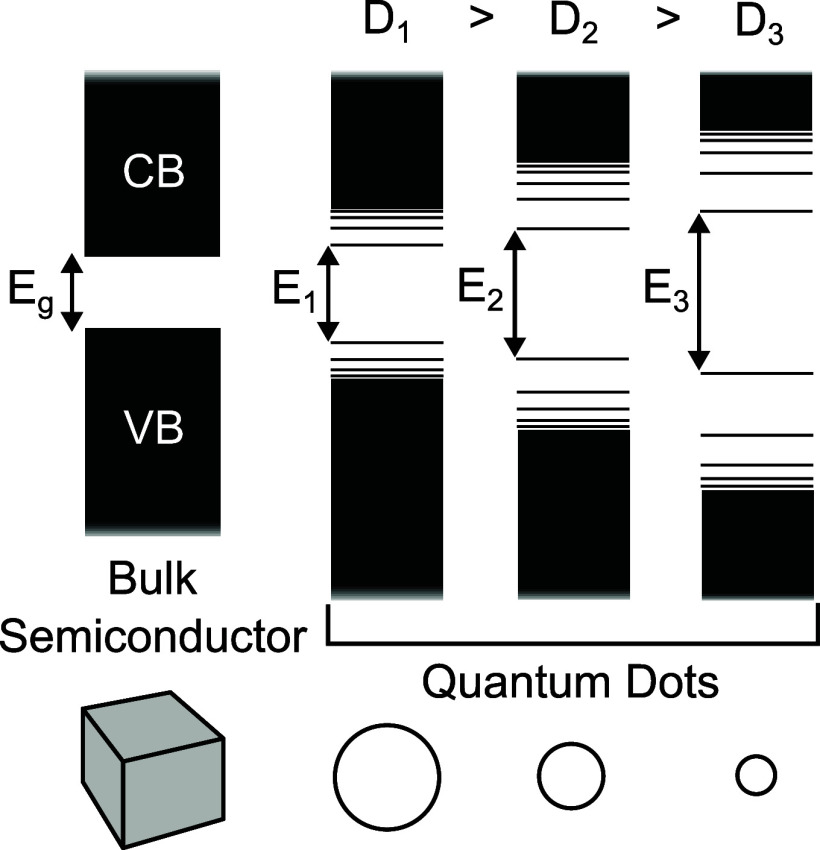
Left: in a bulk semiconductor, the valence band (VB) and conduction band (CB) are separated by the band gap energy *E_g_*. With decreasing quantum dot diameter (*D*_1_ > *D*_2_ > *D*_3_), the ground-state exciton energy increases: *E*_1_ < *E*_2_ < *E*_3_. Both hole levels near the VB and electron levels near the CB are discrete. Adapted from ref 16 Available under a CC-BY license. Copyright 2015 Dong, Wang, Chen, Pan, and Qiu.

The choice of a certain sample of quantum dots for one’s favorite application is crucial since the emission spectrum depends not only on the size but also on the material backbone. Jointly with many teams worldwide, it is our research goal to pursue active silicon nanophotonics using quantum emitters.^17,18^ Therefore, we develop 3D silicon photonic band gap crystals and place quantum dot emitters deep inside the nanostructure to explore advanced quantum optics of a 3D photonic band gap.^19–22^ Since silicon nanostructures have a transparency range limited to 1.1 eV due to the electronic band gap, quantum dots preferably emit below 1.1 eV to avoid absorption, hence our choice toward PbS or lead selenide (PbSe) quantum dots.^4^ We decided to perform our studies with PbS quantum dots as they are commercially available with a variety of organic ligands that allow to attach quantum dots at targeted positions inside a nanophotonic structure.^23^ Furthermore, the organic ligand have a positive effect on the quantum efficiency of the nanocrystal and can stabilize or protect the inorganic core, which is feasible for applications as a single photon quantum source in the telecom range.

Figure 2 shows that the band gap of PbS quantum dot nanocrystals decreases with increasing diameter, as expected. Moreels et al. compiled several data sets of experimental and theoretical data,^24^ where the band gap is determined from optical absorbance spectra and the diameter from electron microscopy. The experimental data from Moreels et al. agree well with those of Cademartiri et al.^25^ and with those of Borrelli and Smith,^26^ even though the latter are different nanocrystals embedded in glass. Since the diameter of the nanocrystals is less than twice the PbS exciton Bohr radius (R_Bohr_ = 18 nm), the exciton wave functions are quantum confined,^28,29^ leading to PbS nanocrystal band gaps exceeding the *E*_*g*_ = 0.41 eV bulk PbS band gap, in agreement with expectation, see Figure 1. The experimental observations also agree well with tight-binding calculations of both Moreels et al. and Kane et al.^27^ From all data combined, Moreels et al. derived a sizing curve that yields the nanocrystal diameter from the optically measured band gap.

**Figure 2:**
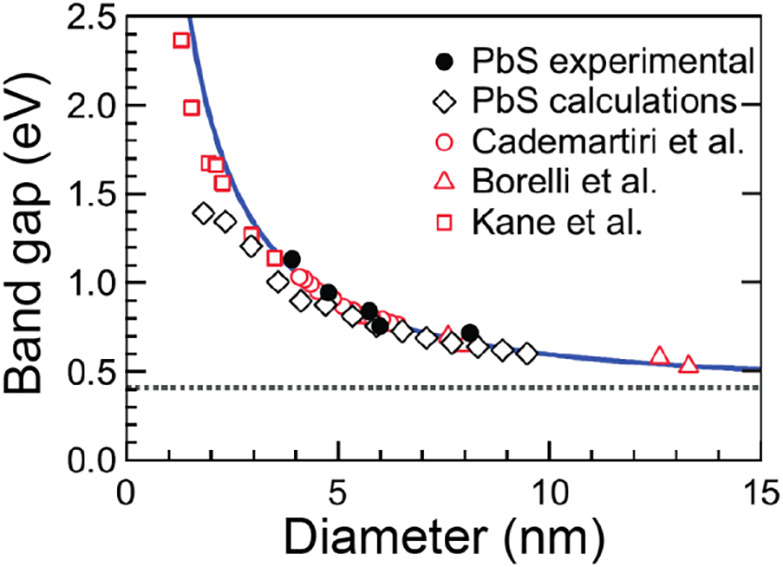
Relation between the PbS quantum dot band gap and the nanocrystal diameter. Adapted from Moreels et al.^24^ Copyright (2009) American Chemical Society. Black circles are measurements from Moreels et al., red circles from Cademartiri et al.,^25^ red triangles from Borrelli and Smith.^26^ Black diamonds are calculations of Moreels et al., and red squares from Kane et al.^27^ The drawn curve is a sizing curve matched to all experimental and theoretical data, and the horizontal dotted line the bulk PbS band gap *E_g_* = 0.41 eV.

To the best of our knowledge, however, there are no systematic studies of the *emission properties* of NIR quantum dots, in particular of the emission rate versus emission photon energy or equivalently (and inversely) versus quantum dot diameter. In contrast, the literature cited above typically describes absorption properties, which characterizes only part of the optical properties. Commercial PbS quantum dots were found to be available with broad emission in the range 0.8 to 1.4 eV corresponding to wavenumbers 6450 < *ν̃* < 11500 cm^–1^, or wavelengths 880 < λ < 1550 nm. Since we study samples of PbS quantum dots from different suppliers, we characterized different QDots whose emission range matched with the 3D photonic band gap of our photonic crystals.^18,23^ For the purpose of dynamic time-resolved quantum optics and quantum information devices,^30^ we characterize the QDot emission dynamics. From the combined data we aim to infer general behavior of PbS nanocrystal quantum dots.

## Methods

### Optical Setup

The setup that was used to collect the emission spectra and frequency-resolved time-resolved emission measurements is described in detail previously.^18^ In brief, we use a pulsed diode laser (wavelength λ ≈ 685 nm, PicoQuant LDH-C) with a low pulse repetition rate 62.5 kHz and low power of 9.7 mW to excite the QDots in the single exciton regime.^31^ Time-resolved emission is collected with the time-correlated single photon counting (TCSPC) method,^32^ using a indium gallium arsenide (InGaAs) photomultiplier (Hamamatsu H10330–75) attached to a spectrometer (Princeton Instruments PI Spectra Pro 2558) to select the emission frequencies. The sample holder was adapted since the quantum dot suspension were contained in small glass capillaries. In the emission spectrum measurements, we employ an InGaAs diode array (PI OMA-V) that has from several dead pixels (due to earlier inadvertent overexposure), which we blank out, hence some spectra reveal an apparent hole that has no scientific meaning.

### Quantum Dot Nanoparticles

We study PbS nanoparticle quantum dots, whose emission wavelengths are compatible with silicon nanophotonic crystals, that is, the emission wavelength is longer than the Si band gap absorption edge at 1100 nm. The nanoparticles were obtained in aqueous suspension from Suzhou Xingshuo Nanotech Co., Ltd. (Mesolight), and in toluene suspensions from Evident Technologies, from M K Impex Corp. (MKNano), and from STREM Chemicals, Inc. (CANdots). The PEG-coated quantum dots were stored in a refrigerator at 4°C, while the rest was kept in a glovebox (MBRAUN LABstar).

## Results and Discussion

### Emission Spectra and Nanocrystal Dimensions

We present in Figure 3 the continuous-wave (cw) emission spectra obtained from all different quantum dot suspensions. We observe a broad spectral distribution for each of the quantum dot suspensions compared to quantum dots that emit within visible wavelengths (fwhm Δλ = 12–42 nm),^33^ but within acceptable values (fwhm Δλ = 100–160 nm) compared to near-infrared emitting PbS quantum dots.^34^ The spectral width is attributed to the size distribution of the quantum dots, due to the statistical nature of the liquid synthesis following the pioneering work of Murray et al.^35^ Table 1 lists emission peak wavelengths λ_max_, emission peak wavenumbers *ν̃*_max_, full width at half-maximum (fwhm), that are all in the range of silicon transparency, and also cover several telecom bands.^36^

**Figure 3:**
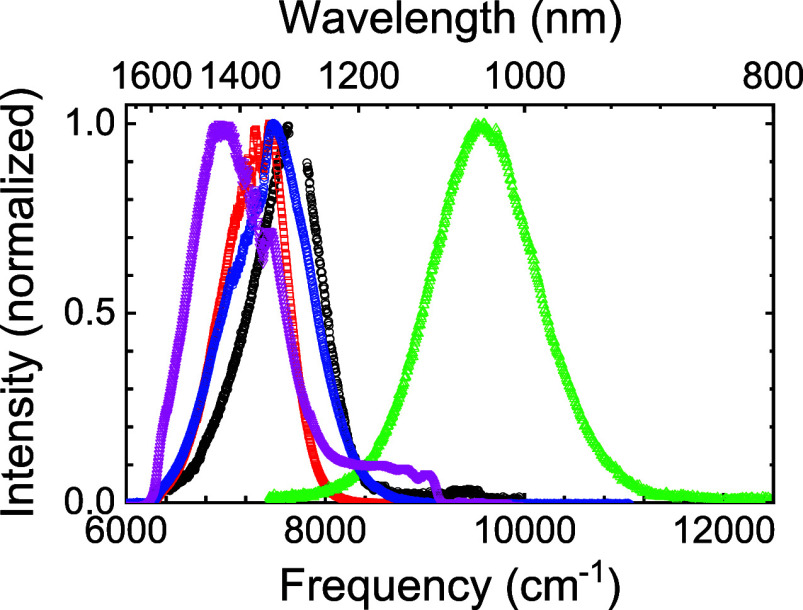
Scaled emission spectra measured for all quantum dot suspensions, bottom abscissa gives wavenumbers and the top one wavelengths. Blue data are for sample A (times 1.64), Black data are for QDot sample B (times 4.34), magenta data are for sample C, green data for sample D (times 4.03), and red data for sample E (times 1.02).

**Table 1:** Properties of the Quantum Dots (QDs) Studied in This Paper^a^

QD	Origin/surface/solvent	*ν̃*_max_	λ_max_	fwhm	Δλ/λ	Diameter
A	CANdots/OA/toluene	7486 cm^–1^	1336 nm	172 nm	12.9%	3.82–6.19 nm
B	Evident/OA/toluene	7684 cm^–1^	1301 nm	136 nm	10.5%	3.96–6.15 nm
C	Mesolight/PEG-NH_2_/CHCl_3_	7037 cm^–1^	1421 nm	210 nm	14.8%	4.14–6.19 nm
D	Mesolight/PEG-NH_2_/H_2_O	9514 cm^–1^	1051 nm	133 nm	12.7%	2.81–4.46 nm
E	MKNano/OA/toluene	7434 cm^–1^	1345 nm	138 nm	10.3%	4.15–6.17 nm

a The 1st column gives the label, 2nd column the origin, surface group and solvent (OA is oleic acid, PEG polyethylene glycol), 3rd column wavenumber maximum, 4th column corresponding wavelength maximum, 5th column wavelength FHWM, 6th column relative bandwidth, 7th column diameter where the range corresponds to the FWHM.

From the emission spectra, we infer the quantum dot diameters and the concomitant size distribution, starting from the sizing curve of Moreels et al.^24^ Whereas Moreels et al. used the optical absorption peak to estimate the exciton energy, we use here the peak in the emission spectrum to gauge the exciton. Therefore, to reasonably invoke the Moreels et al. sizing curve, we must assume that the Stokes shift is small. Indeed, this assumption is validated by the discussion of Husken^31^ on similar PbSe nanocrystals that reveal a shift of a few 10*s* meV, hence only a few % of the exciton energy.

The nanocrystal size distributions listed in Table 1 are conservatively estimated as the ranges where the emission intensities have dropped to 5% of the peak intensities. In the evaluation of sample C, we neglect the unusual square shoulder in the range between 8330 and 9150 cm^–1^ (λ = 1093–1200 nm), and assume that the “true” bell-shaped exciton spectrum ends around *ν̃* = 8330 cm^–1^ (λ = 1200 nm). Since all studied nanocrystal samples have diameters D_size_ ≤ 6.2 nm, we conclude that all quantum dot samples easily fit within the pores of our 3D silicon inverse woodpile photonic crystals that have diameters in excess of D_pore_ ≥ 150 nm, hence it should be readily feasible to dope such photonic crystals with these quantum dots, which is confirmed by our successful previous results.^18,23^

Figure 4 shows a transmission electron microscope (TEM) image of PbS quantum dots capped with thiol–PEG–NH_2_ ligands. A suspension of QDots was placed on a TEM grid to achieve well-dispersed individual nanocrystals on the surface. Individual QDots are highlighted with yellow circles for visualization. In the inset (taken from the bottom left of the overall image and shown enlarged in the top right), individual atoms can be observed at a closer look. The average diameter of the quantum dots is approximately 5 nm.

**Figure 4:**
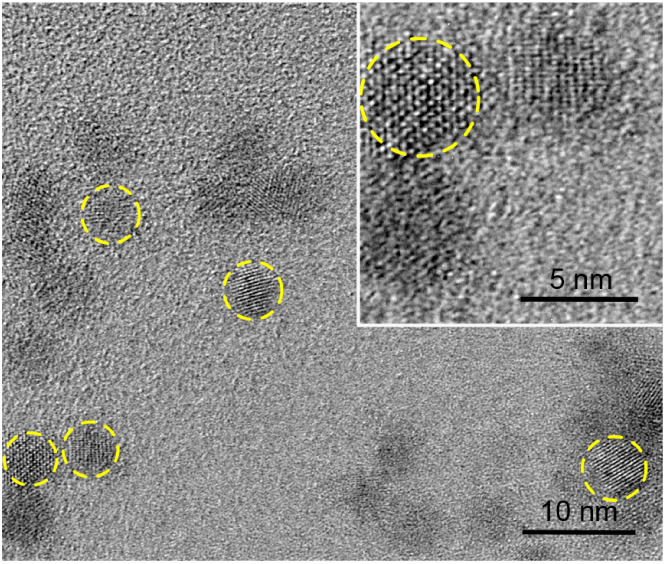
Transmission electron micrograph (TEM) of PbS–PEG–NH_2_ QDots (sample D). Yellow circles mark the position of individual QDots. The scale bar represents 10 nm. Inset: zoom-in of two PbS quantum dots revealing crystal planes. Scale bar represents 5 nm.

### Time-Resolved Emission

Figure 5 shows a typical decay curve of lead sulfide quantum dots with an OA capping suspended in toluene. The decay curve is single-exponential, which means physically that all nanocrystals emit photons with the same total rate Γ_tot_ that is equal to the sum of radiative Γ_rad_ and nonradiative Γ_nonrad_ rates: Γ_tot_ = Γ_rad_ + Γ_nonrad_. While either or both the radiative and the nonradiative rates may be distributed, it seems very unlikely that a distribution of radiative rates would exactly cancel a distribution of nonradiative rates to yield a total decay rate that would have a narrow distribution.

**Figure 5:**
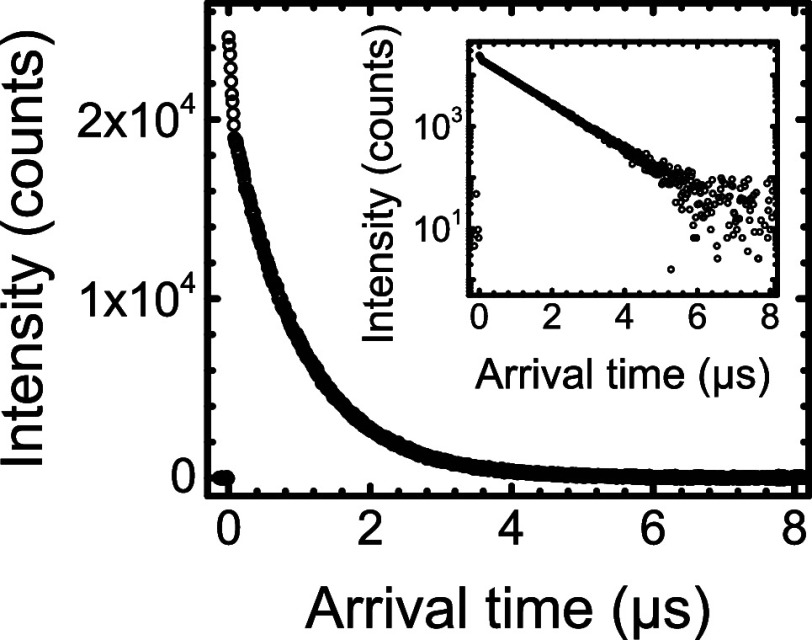
Luminescence decay curve from PbS quantum dots (sample A) at 7463 cm^–1^ (λ = 1340 nm) near the emission peak. Background-corrected counts are shown versus photon arrival time. Inset: the same data on a semilogarithmic plot.

To interpret emission rates from time-resolved decay curves (as in Figure 5), proper care must be taken of the modeling procedure, e.g., refs 17 and 37 (notably the supplementary). Figure 6a shows an example of a nearly exponential decay curve, that is readily modeled with a textbook single-exponential model, yielding a total emission rate $\Gamma_{\mathrm{tot}}^{s}=1.0173\;\mu s^{-1}\pm 0.0003\;\mu s^{-1}$ . In this paper, we report 95% confidence intervals, see refs 17,31 The residuals, shown in the lower panel, are distributed around zero, which is a second indication that the single-exponential is a reliable model. Third, the goodness-of-fit, expressed as a reduced $\chi_{\mathrm{red}}^{2}$ ,^38^ is equal to $\chi_{\mathrm{red}}^{2}=1.02$ , close to the ideal value $\chi_{\mathrm{red}}^{2}=1$ which indicates that the data are reliably described by ubiquitous the single-exponential decay model.^39^ Hence, at this emission wavenumber, all OA-covered quantum dots in toluene in sample A have closely the same excited-state lifetime.

**Figure 6:**
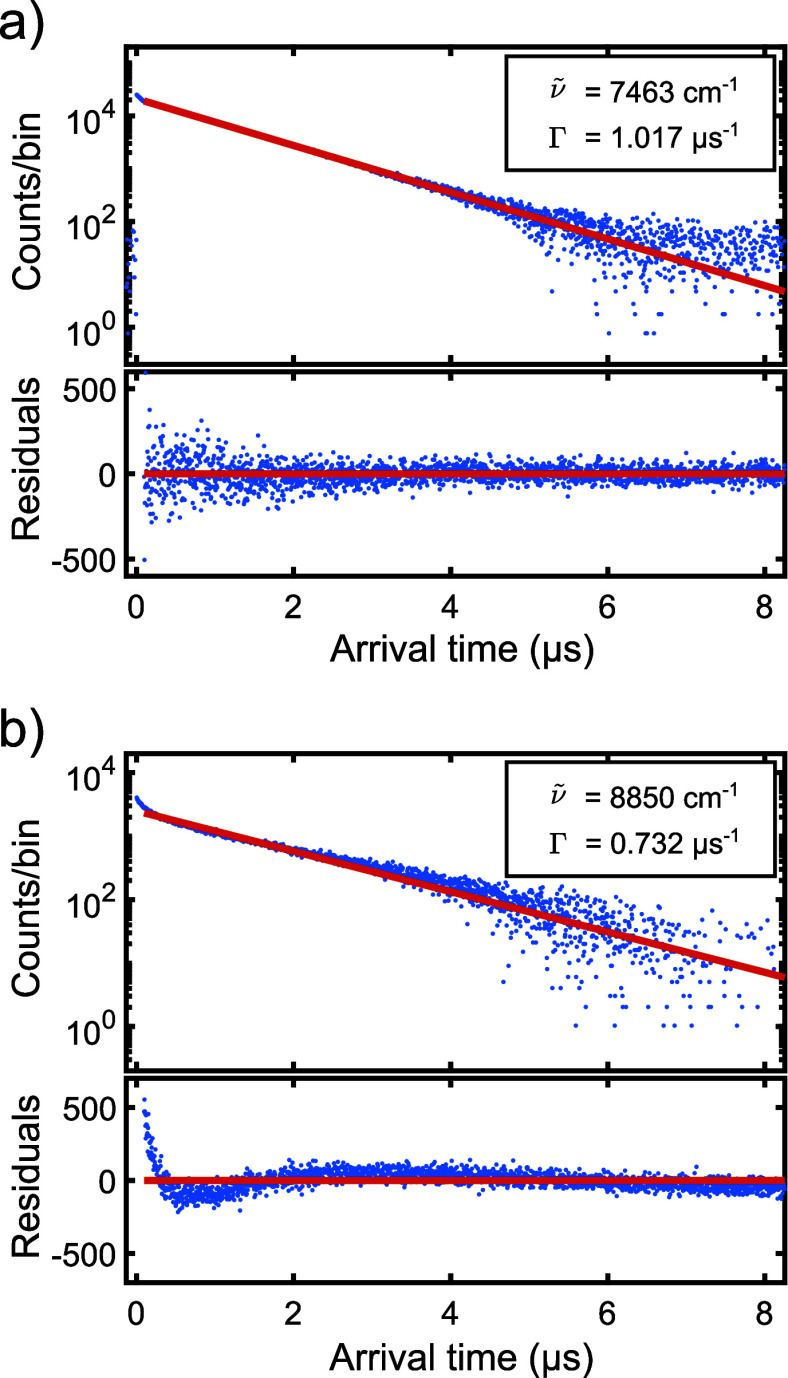
Examples of single exponential and nonsingle exponential decay histograms. The legends list the decay rate of the single-exponential model and the lower panels show the residuals between data and model. (a) Histogram for sample A at *ν̃* = 7463 cm^–1^ (λ = 1340 nm) that agrees well with a single-exponential decay model since the residuals are randomly distributed about zero. (b) Histogram for sample D at *ν̃* = 8850 cm^–1^ (λ = 1130 nm) that differs markedly from single-exponential decay since the residuals systematically deviate from zero.

Figure 6b shows an example of a decay curve from PEG-NH_2_-capped PbS quantum dots suspended in water that markedly deviates from exponential decay. The data differ from the single-exponential model notably at short and at long arrival times. Indeed, the residuals for the single-exponential model are systematically nonzero at short arrival times below 0.5 μs^–1^, but also in the whole time domain, indicative of a nonmatching model. Third, the goodness-of-fit is equal to $\chi_{\mathrm{red}}^{2}=2.45$ which differs markedly from 1. Physically, such a nonexponential decay curve means that the quantum dots have a distribution of total rates Γ_tot_, as a result of a distribution of nonradiative rates Γ_nonrad_.^37^ [Fn fn1]

Since nonsingle exponential decay is often observed with quantum dot nanocrystals,^41–44^ much work has been done to practically model a physically significant distribution of decay rates to a decay curve in a proper statistical way, see refs 37,45 , and^46^. A time-resolved decay histogram of photon arrival times *t* is described as

1 $$I\left(t\right)=I\left(0\right)\int_{\Gamma =0}^{\infty }\phi \left(\Gamma \right)e^{-\Gamma_{tot}t}d\Gamma$$

where ϕ­(Γ) represents a probability distribution of single-exponential decays over total decay rates Γ with dimension of inverse decay rate. In eq 1, each fraction of quantum dots (corresponding to an infinitesimal interval dΓ) emits single exponentially, and a sum (or integral) of different single exponential decays yields a multiexponential decay, that appears curved in a semilog plot, as in the example in Figure 6b. A particularly successful probability distribution was found to be a log-normal distribution (also known as Schulz distribution^47^ of total decay rates ϕ­(Γ), in other words, a Gaussian plotted on a logarithmic abscissa:^37,45^

2 $$\phi \left(\Gamma \right)=A\exp\left[-{\left(\frac{\ln\Gamma -\ln\Gamma_{mf}}{w}\right)}^{2}\right]$$

where *A* is a normalization constant, Γ_*mf*_ the most frequent decay rate (see Figure 7), and *w* is a dimensionless width parameter that determines the distribution width ΔΓ at $\phi =\frac{1}{e}$

3 $$\Delta \Gamma =2\Gamma_{mf}\sin\;h\left(w\right)$$

Since the amplitude *A*, the most frequent rate constant Γ_*mf*_, and *w* are adjustable parameters, there is only one additional adjustable parameter compared to a single-exponential model. While the model is heuristic (”it fits well with few parameters”) the log-normal shape has several rationales, namely that a nanocrystal size distribution is typically also log-normal or Schulz distributed,^47^ the log-normal distribution only allows positive rates as it should physically be.[Fn fn2] Since a log-normal model has only one more adjustable parameter compared to a single-exponential decay model, it is numerically stable.

**Figure 7:**
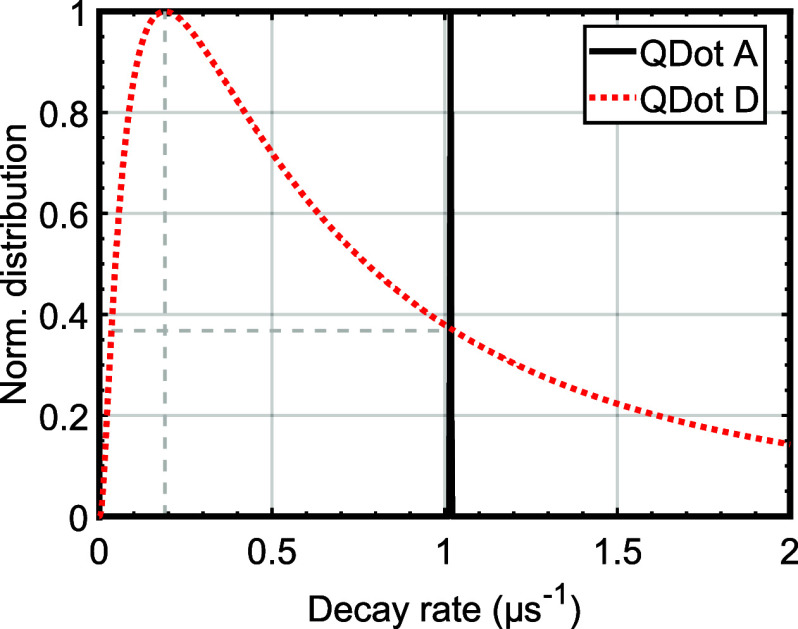
Examples of log-normal distributions of decay rates σ­(Γ_tot_). The dotted red curve is for parameters Γ*_mf_* = 0.109 and ΔΓ = 0.991 from Figure 8b, and the black curve is for parameters Γ*_mf_* = 1.017 and ΔΓ = 0.003 from Figure 8a.

In Figure 8, we show the same two exemplary decay histogram as in Figure 6, now modeled with log-normal distributions of total decay rates. Sample D reveals a clear nonexponential decay, that is much better modeled with the log-normal distribution. Indeed, the goodness-of-fit improves to $\chi_{\mathrm{red}}^{2}=1.32$ , much closer to 1 than for the single-exponential model $\left(\chi_{\mathrm{red}}^{2}=2.45\right)$ . The resulting most-frequent decay rate is Γ_*mf*_ = 0.109 μs^–1^ and the width is ΔΓ = 0.991 μs^–1^, hence the width is much greater than the most frequent rate (ΔΓ > Γ_*mf*_), characteristic of a broad distribution of total rates, see Figure 7. From the log-normal model for the nearly exponentially decaying CANdots, we obtain a decay rate Γ_*mf*_ = 1.017 μs^–1^ with a goodness-of-fit χ_*red*_^2^ = 1.0191. The width is ΔΓ = 0.003 μs^–1^, much less than the most frequency decay rate, typical of a narrow distribution, as shown in Figure 7. These results show that the log-normal model successfully describes the time-resolved decay of NIR and SWIR (short-wave infrared) emitting colloidal quantum dots, including with different cappings and in different solvents.

**Figure 8:**
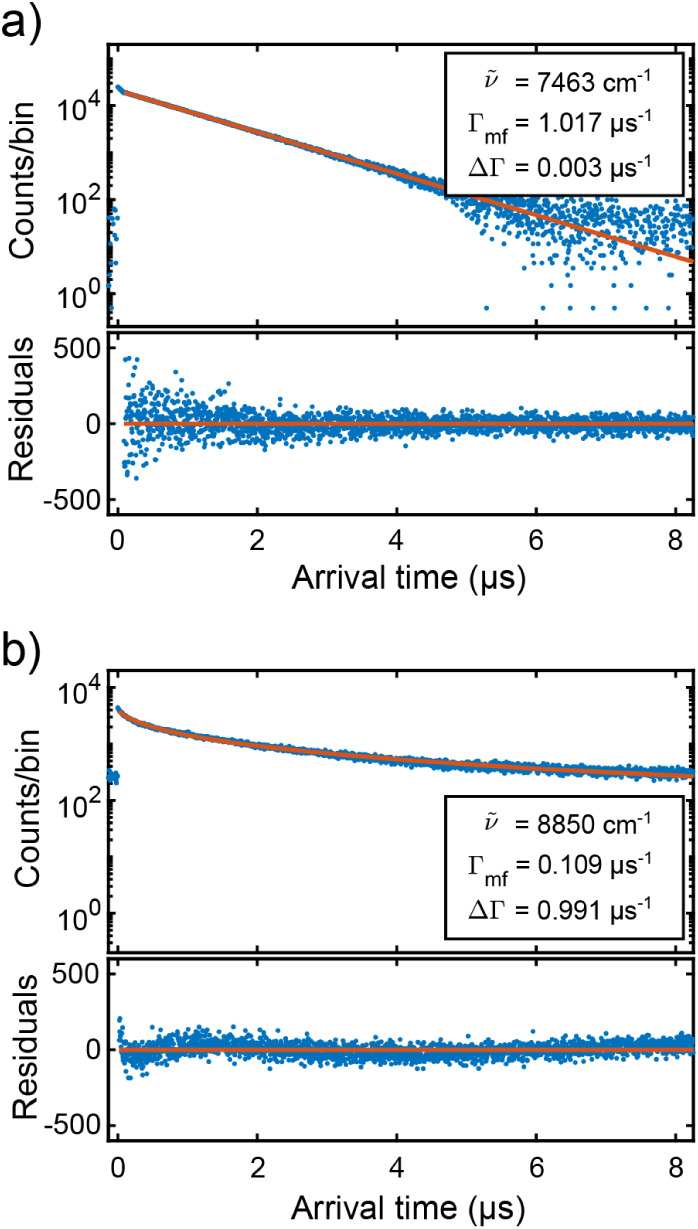
Examples of a) sample A b) sample D with a log-normal distribution of decay rates^37^ that are seen to yield small residuals centered about 0.

### Emission Rate versus Frequency and Size

Figure 9a shows the decay rate Γ versus both wavenumber (bottom abscissa) and wavelength (top abscissa), specifically the most frequent decay rate Γ_*mf*_ obtained from the log-normal models.

**Figure 9:**
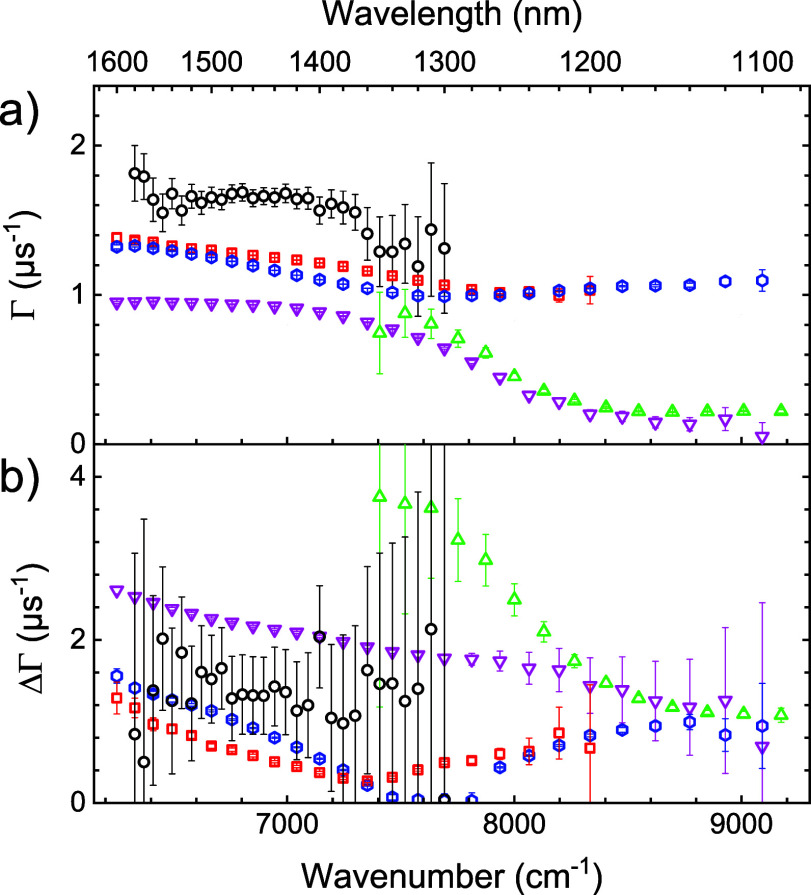
Measured decay rates including error bars for all quantum dot suspensions versus wavenumber (bottom abscissa) or wavelength (top abscissa). (a) Most-frequent decay rates Γ*_mf_* from the log-normal model, blue hexagons represent sample A (OA, toluene), black circles sample B (OA, toluene), and magenta inverted triangles sample C (PEG, chloroform), green triangles sample D (PEG, water), and red squares sample E (OA, toluene). (b) Logarithmic widths of the distribution ΔΓ from the log-normal model, symbols same as in (a).

If we first zoom in on the OA-capped quantum dots suspended in toluene (samples A, B, E), we see that overall the decay rate is fairly constant with frequencies over a broad range from 6200 to 9100 cm^–1^ which suggests that the emission oscillator strength is independent of the quantum dot diameter. In detail, at low frequency below 7500 cm^–1^ there is a mild decreasing trend and above 8000 cm^–1^ a slightly increasing trend, but in view of sample to sample variations and other sources of uncertainty it is not certain that these trends are significant. The PEG-capped quantum dots (samples C, D) also show a constant (or slightly decreasing) trend around 1 μs^–1^ up to about 7200 cm^–1^. But with further increasing frequency the emission rate decreases dramatically by nearly 5-fold to about 0.2 μs^–1^ up to about 8500 cm^–1^ and remaining constant at higher frequency. This sudden decrease is striking as it would correspond to a rapid and counterintuitive decrease of the oscillator strength with increasing quantum dot diameter.

Figure 9b shows the logarithmic widths of the decay rate distributions ΔΓ versus wavenumber (bottom abscissa) and wavelength (top abscissa). First concentrating on the OA-capped quantum dots suspended in toluene (samples A, B, E), the widths start at ΔΓ = 1.2 to 1.6 μs^–1^, decrease with frequency up to 7300 cm^–1^ (sample E) or 7500 cm^–1^ (sample A, close to the spectral maxima, see Table 1) to nearly vanishing width, characteristic of single-exponential decay. Subsequently, the widths slightly increase to about ΔΓ = 1.0 μs^–1^. For sample B, the widths vary between ΔΓ = 1 and 2 μs^–1^ with a relatively large uncertainty so it is not reasonable to assign a trend. The PEG-capped quantum dots (samples C, D) show monotonously decreasing trends over the whole accessible frequency ranges, ranging from ΔΓ = 2.5 to 1.6 μs^–1^ (sample C) or from ΔΓ = 3.8 to 1.1 μs^–1^ (sample D). Overall, it is also clear that all OA-capped quantum dots have (much) narrower decay distributions than the PEG-capped quantum dots.

### Sharply Decreasing Emission Rates with PEG Surface Groups

We have seen in Figure 9a that quantum dots with PEG capping have a dramatically different trend (above 8000 cm^–1^) than the ones capped with oleic acid. Whereas quantum dot emission frequency parametrizes the optical properties, it is in this discussion also relevant to consider the quantum dot’s physical chemical properties as parametrized by diameter. Therefore, we plot in Figure 10a and b the same data as in Figure 9, yet versus quantum dot diameter, as obtained from the emission frequency with the sizing curve of Moreels et al.^24^ (see Figure 2). For the OA-capped PbS quantum dots in toluene, we observe in Figure 10a a slight decrease of the most frequent rate Γ_*mf*_ with increasing diameter, followed by a slight increase above D_size_ = 4.4 nm. For both PEG-capped PbS quantum dots in both water and chloroform, we see that the most frequent rate Γ_*mf*_ increases markedly in a remarkable S-like shape centered near D_size_ = 4.4 nm.

**Figure 10:**
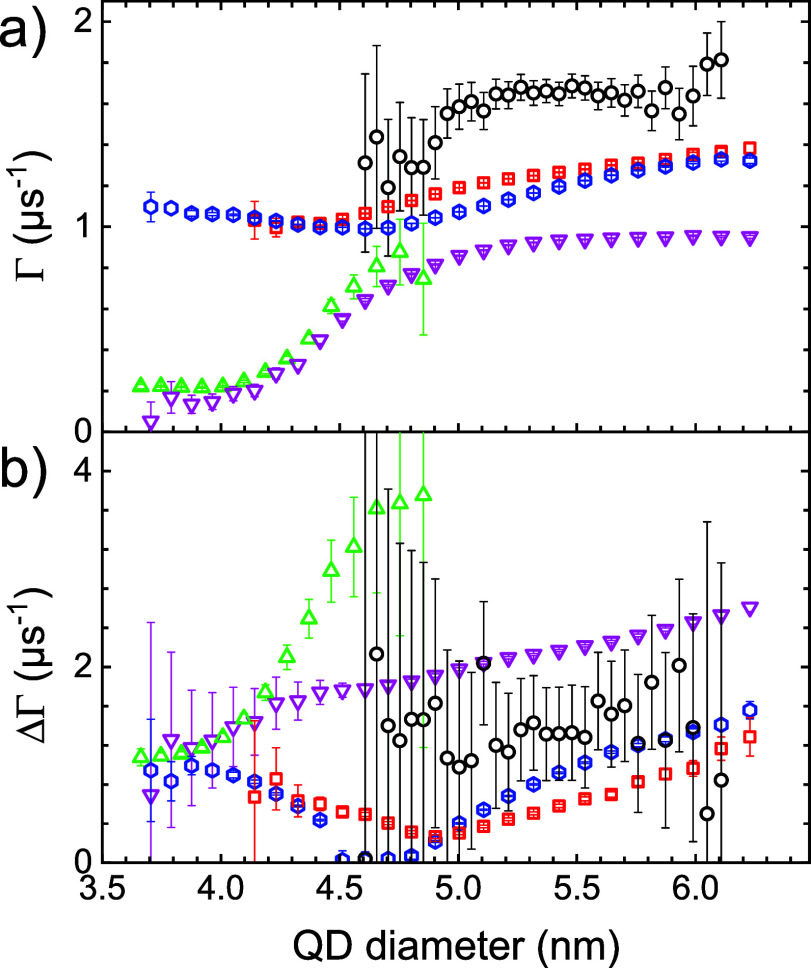
Measured quantum dot decay rates versus quantum dot (QD) diameter, obtained from modeling the decay curves with a log-normal model. (a) Most frequent decay rate Γ*_mf_*, (b) logarithmic width of the distribution ΔΓ derived from fitting with log-normal. The blue hexagons represent sample A (OA, toluene), black circles sample B (OA, toluene), magenta inverted triangles sample C (PEG, chloroform), green triangles sample D (PEG, water), and red squares sample E (OA, toluene).

A reasonable hypothesis for the dramatic decrease in decay rates is the enhanced local electric field generated by the thiol–PEG–NH_2_ ligands, that does not arise from solvent molecules or OH^–^ adsorption at the PEG–water interface. During the ligand exchange process, the native oleic acid ligands are replaced by thiol–PEG–NH_2_.^48–50^ The thiol headgroup binds as thiolate (S^–^) to undercoordinated Pb sites on the QDot surface, contributing a negative charge near the inorganic core. However, we assign an even stronger local electric field effect to the terminal −NH_2_ group. In aqueous media (in our case deionized water at pH 7) the −NH_2_ group is largely protonated to $-{\mathrm{NH}}_{3}^{+}$ , creating a net positive charge at the outer periphery of the PEG brush, while in chloroform the neutral but highly polar −NH_2_ still produces a significant surface dipole. Because smaller PbS QDots possess a more Pb-rich surface and a higher surface-to-volume ratio,^24^ this positive/polar outer shell induces a stronger inward-directed radial electric field throughout the nanocrystal core. Combined with tighter quantum confinement in smaller QDots, the field results in a more pronounced quantum-confined Stark effect (QCSE). This, in turn, leads to a greater spatial separation of the electron and hole wave function in smaller QDots, which reduces the wave function overlap and reduces the radiative recombination rate.^51–55^

Let us briefly mention four additional hypotheses for the striking decrease. First, it is from the outset remarkable that the major trend is a *decrease* of the emission rate, since this rate represents a *total* emission rate that is the sum of the radiative and nonradiative rates. Hence, common quenching mechanisms (like charge transfer, energy transfer, see ref 39) may safely be excluded, since all of these would yield an *increase* of the nonradiative rate, which contradicts our observations. The decrease in total emission rate does not necessarily signify a decrease of the quantum yield. On smaller PbS QDots, the more highly curved surface and higher Pb-rich character often allow more effective passivation of nonradiative trap states (such as undercoordinated Pb sites or dangling bonds) by the thiol–PEG ligands. This can suppress nonradiative recombination pathways more strongly than on larger QDots, even if the radiative decay rate itself is modestly reduced due to intrinsic nanocrystal size effects.

A second additional hypothesis is to invoke a physicochemical reason for the suspending liquid, notably the polarity and proticity of the solvent. We deem this hypothesis not likely, since the dramatic decrease occurs both for QDots suspended in water (polar, protic) and in chloroform (weakly polar, aprotic).

A third additional hypothesis is whether there is a resonance in the suspending liquid that might ″siphon off″ the excitation from the quantum dot and reduce the total emission rate. Apart from the problem that this hypothesis corresponds to an increased total emission rate at variance with observations, another problem with this hypothesis is that there are no known strong resonances above 8000 cm^–1^ in either water and chloroform, since vibrational resonances are below 3600 cm^–1^ (the well-known O–H stretch in water) and electronic resonances are above 25000 cm^–1^; in other words, both water and chloroform are completely transparent in the relevant range above 8000 cm^–1^.

A fourth additional hypothesis is that an acoustic (surface) mode is excited by the presence of the excited electron–hole pair in PEG-covered QDots, as reported on thin films and nanoparticles,^56–58^ where THz-range oscillations were reported. This hypothesis also does not match with our observations since the resonance frequencies of acoustic oscillations are at least 20-fold too low compared to the relevant range above 8000 cm^–1^, and is thus also falsified.

### Interpretations of Frequency-Dependent Decay Rates

To interpret the broadband frequency-dependent decay rates of the PbS quantum dot nanocrystals, we replot in Figure 11 the data from Figure 9a, where we restrict this discussion to the relative simple behavior of the OA-capped quantum dots suspended in toluene, and compare the measurements to several different theoretical models.

**Figure 11:**
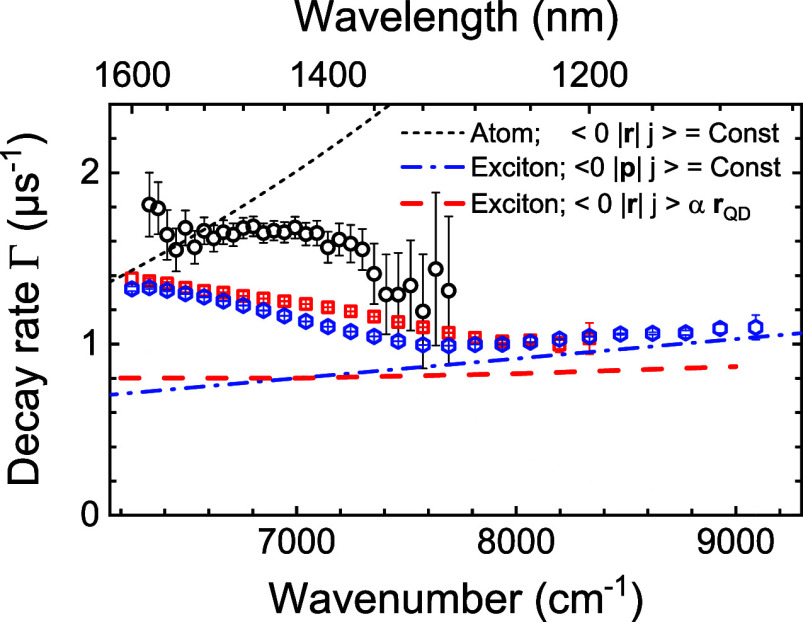
Most-frequent decay rates Γ_*mf*_ from the log-normal model. The black dotted line represents a model of an atomic dipole, the blue dashed-dotted line an ideal two-level exciton with constant transition dipole, and the red dashed curve an ideal two-level exciton with transition dipole proportional to quantum dot diameter.

First, let us consider a quantum-mechanical oscillator typical of a two-level atomic emitter,^59^ since quantum dots were originally advertised to be “solid-state atoms”,^2^ and since this model was employed in early nanophotonic work.^60^ In a homogeneous dielectric medium with refractive index *n*_*m*_, the radiative emission rate Γ_at_ as a function of (angular) emission frequency ω is equal to

4 $$\Gamma_{\mathrm{at}}\left(\omega \right)=\frac{\omega^{3}n_{m}\mu_{if}}{3\pi \epsilon_{0}\hbar c^{3}}$$

with μ_*if*_ the transition dipole moment, ϵ_0_ the dielectric permittivity of free space, *ℏ* the reduced Planck’s constant, *c* the speed of light in free space. The resulting curve is plotted in Figure 11, where we assume a refractive index *n*_*m*_ = 1.4338 for toluene, and a transition dipole moment equal to |μ_*if*_| = 3.6 D.^61^ The model does not agree with the data since its trend is much too steep. Therefore, we conclude that PbS quantum dots as quantum emitters are not behaving like artificial atoms, thereby matching the original warning of Petroff et al.^2^

As a second model, we consider the radiative emission rate from a single exciton Γ_ex_ (excited electron–hole pair) to the ground state where the particles have recombined. Following refs 62,63 the rate is equal to

5 $$\Gamma_{\mathrm{ex}}\left(\omega \right)=\frac{ne^{2}F^{2}}{3\pi \epsilon_{0}m^{2}\hbar c^{3}}\omega \left|\left\langle 0\right|p\right|j\rangle {|}^{2}$$

with *e* the electron charge, *F* the local field factor (of order unity), *m* the electron rest mass, and ⟨0| ***p*** |*j*⟩ the transition dipole moment in momentum ***p*** space (instead of in position ***r*** space as is usual with atoms). Assuming that interband transitions are mostly determined by the Bloch functions in the unit cell, the momentum-space transition dipole moment is *independent* of the nanocrystal diameter or the emission frequency, hence the decay rate of an ideal two-level exciton is linearly proportional to the emission frequency, as predicted in earlier work.^10^ The rate of the ideal two-level exciton model is shown in Figure 11, where we take |⟨0| ***p*** |*j*⟩| = 3.6 D from theoretical work of Liu et al.^61^ The model agrees better with our observations than the dipolar atomic model, in particular, the ideal two-level exciton model agrees very well at frequencies above 7600 cm^–1^ both in the magnitude and in the trend. At frequencies below 7600 cm^–1^ the model agrees less well with the data, notably since the data reveal a *decreasing* trend that is opposite to the *increasing* trend of the model. Nevertheless, the transition dipole moment of 3.6 D gives a good agreement with the observations (assuming all measured decay to be radiative), which means that the transition dipole of PbS nanocrystal quantum dots is substantial as it is greater than, e.g., CdSe nanocrystal quantum dots.^64^

As a third model, also shown in Figure 11, we heuristically modify the ideal two-level exciton model by assuming the transition dipole moment (in momentum space) not to be constant, but that the magnitude of the dipole moment is proportional to the radius of the nanocrystals |⟨0| ***p*** |*j*⟩|∝(D_size_/2). Since we can only assume a proportionality, we have to fix the offset, which we choose (somewhat arbitrarily) at 7000 cm^–1^. It is clear that the trend of this model is in better overall agreement than the ideal two-level model, it also does not explain the slightly downward trend in the observations over the whole range from 6200 to 9200 cm^–1^. Therefore, more theoretical work is called for to model the size and frequency-dependent emission rate of PbS nanocrystal quantum dots.

To place our observations in perspective, we briefly discuss previous work on different quantum dots, both nanocrystalline and self-assembled ones. Van Driel et al. studied CdSe and CdTe nanocrystal quantum dots in suspension and observed markedly increasing trends of the total decay rate Γ_*tot*_ with frequency,^62,63^ which was successfully described by the thermal occupation of dark exciton states at room temperature that give rise to a strong decrease of the emission rate, and a supra-linear trend that matched well with tight-binding calculations. Walters et al. studied Si nanocrystals fabricated by ion implantation at a variable distance to a reflector to distinguish radiative from nonradiative decay (by the Drexhage effect^40,65^ and reported a radiative radiative rate Γ_rad_ that increases with frequency.^66^ Johansen et al. studied self-assembled InAs quantum dots made by molecular beam epitaxy (MBE) by the Drexhage effect and reported that the radiative rate Γ_rad_ slightly decreases with increasing frequency, which was successfully explained by a decreasing oscillator strength with decreasing quantum dot size, due to a concomitant decreasing overlap of the electron and hole wave functions.^13^ Leistikow et al. studied CdSe quantum dots by the Drexhage effect and observed that the radiative rate Γ_rad_ first increases and then decreases with increasing frequency.^64^ Thus, the trends in Figure 9a for OA-capped quantum dots in toluene are similar to those for self-assembled InAs quantum dots. In contrast, the strongly varying decay rates of the PEG-capped quantum dots differ strikingly from all earlier observations reviewed above.

### Quantum Dots on a Silicon Surface

Figure 12 shows the frequency-dependent emission rates of lead sulfide quantum dots dip-coated on a flat silicon surface. We observe an increase of the decay rates with increasing wavenumber which is expected from our ideal two-level exciton model.

**Figure 12:**
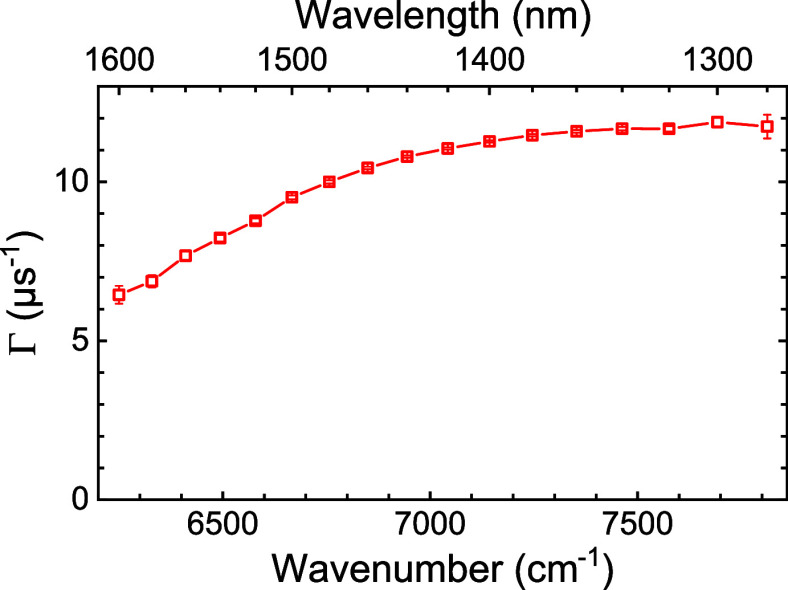
Frequency dependent excited-state lifetime measurements of sample E quantum dots dip-coated on a clean silicon bar. The decay rates are plotted versus wavenumber (bottom abscissa) and wavelength (top abscissa).

For the dip-coating procedure we used MKNano PbS-OA quantum dots (red) from Figure 9. The decay rates increased from ≈1.1 μs^–1^ to ≈ 10 μs^–1^. The increase is attributed to the quantum dots being in close vicinity to a medium (silicon) with a high ϵ at optical frequencies, which is known to enhance the local density of states and hence the rate and thus total emission rates.^40,65^

## Conclusions

We present broadband spectra and excited-state lifetimes of lead sulfide quantum dots with different surface groups (oleic acid and polyethylene glycol) suspended in polar and apolar liquids. The time-dependent emission revealed both exponential and nonexponential decay traces, and all were successfully modeled with a log-normal distribution of decay rates. For OA-covered QDots we find that the most frequent decay rate is nearly constant with frequency, whereas the PEG-covered QDots reveal a remarkable downturn starting at 8000 cm^–1^ (wavelength λ = 1250 nm) or conversely for nanocrystal diameters less than 4.4 nm. We compare our experimental data to several theoretical models. An atomic point dipole does not at all match with the data, an ideal two-level-exciton model gives a reasonable description, which slightly improves if we assume the transition dipole moment to scale linearly with the QDot diameter. For the remarkable PEG–QDot downturn we have discussed five possible hypotheses—namely quenching in general, solvent polarity, generic resonances, acoustic (surface) modes, exciton or charge trapping—none of which convincingly explains the observations, thus calling for future study including theory or simulations. On a dip-coated silicon substrate, we observe an increase of the decay rate with frequency which agrees with expectations from the literature. The knowledge assembled here is relevant to understand the optical properties of lead sulfide quantum dots attached to a polymer layer in- and outside silicon photonic crystals.

## Data Availability

A preprint of this manuscript is available on the ChemRxiv preprint server.^67^

## Footnotes

1 We can safely exclude the possibility that the *radiative* rates are distributed, since the quantum dots are located in a homogeneous dielectric medium with a constant local density of optical states.^40^

2 Other models include Gaussian or Lorentzian distributions of total rates, that have the undesirable feature that they include unphysical negative rates. To remedy this, one may crop the distribution to zero rate, which has the disadvantages that the distribution is not analytical anymore and that proper normalization of the distribution is compromised, see ref 37. Biexponential or discrete multiexponential decay models are not employed as there is no a priori scientific reason for 2 or N decay channels in our system.

## #

Complex Photonic Systems (COPS) group, Photonics and Semiconductor Nanophysics (PSN) Chair, Department of Applied Physics and Science Education (APSE), Eindhoven University of Technology (TU/e), P.O. Box 513, 5600 MB Eindhoven, The Netherlands
